# Proportion and risk factors of diabetic retinopathy by stage in less-developed rural areas of Hunan province of China: A multi-site cross-sectional study

**DOI:** 10.1186/s12889-022-14232-3

**Published:** 2022-10-07

**Authors:** Yao Chen, Yuanyuan Jiang, Xiaoxi Yao, Yimeng Li, Ruisi Liu, Wencong Lv, Qinyi Zhong, Bin Yan, Yongan Meng, Jing Luo, Mengbo Wu, Jia Guo

**Affiliations:** 1 Xiangya School of Nursing, Central South University, 410013 Changsha, Hunan China; 2 Shenzhen College of International Education, 518017 Shenzhen, China; 3 Xiangya School of Public Health, Central South University, 410013 Changsha, China; 4 Department of Ophthalmology, The Second Xiangya Hospital, Central South University, 410011 Changsha, China; 5 Clinical Nursing Teaching and Research Section, The Second Xiangya Hospital, Central South University, 410011 Changsha, China; 6 Nursing School of Xinjiang Medical University, 830054 Urumqi, China

**Keywords:** Type 2 diabetes mellitus, Retinopathy, Proportion, Risk factors

## Abstract

**Aims:**

To investigate the proportion and risk factors of diabetic retinopathy (DR) by stages in less-developed rural areas in Hunan Province of China.

**Background:**

DR is common among people with diabetes but not well recognized in less-developed rural areas. There is insufficient evidence on the risk factors of DR by stages, making it challenging to develop targeted prevention and intervention programs for DR in primary care settings.

**Methods:**

A multi-site cross-sectional survey was conducted among people with type 2 diabetes mellitus (T2DM) from four less-developed counties in Hunan Province of China. All participants underwent the screening of DR via digital fundus photography and completed self-reported questionnaires on their socio-demographic and clinical characteristics, diabetes self-efficacy, diabetes self-care behaviors, social support, family function, and health service accessibility. The multinomial logistic regression models were employed to explore the risk factors of DR by stage, which were selected based on the socio-ecological model, literature, and clinical experience.

**Results:**

A total of 196 participants were included in this study with an average age of 57.43 ± 10.26. 59.6% (117/196) of the participants were identified as DR, including 37.2% (73/196) non-proliferative DR and 22.4% (44/196) proliferative DR. Compared to the non-DR group, the risk factors of non-proliferative DR and proliferative DR were diabetes duration (OR: 1.10, 95 CI%: 1.04–1.17; OR: 1.14, 95 CI% 1.06–1.22) and self-monitoring of blood glucose (OR: 1.09, 95 CI% 1.01–1.17; OR: 1.11, 95 CI%: 1.02–1.20); the protective factors of non-proliferative DR was accessible complication prevention and management education (OR: 0.37, 95 CI% 0.14–0.94) while the protective factors of proliferative DR were physical activities (OR: 0.89, 95 CI%: 0.80–0.98). Compared to the non-proliferative DR group, the protective factors of proliferative DR were physical activities (OR: 0.89, 95 CI% 0.02–0.89) and family function (OR: 0.84, 95 CI%: 0.04–0.84).

**Conclusion:**

DR was prevalent among people with T2DM in less-developed rural areas, indicating the need of strengthening DR screening. Risk factors of DR varied by stage while sharing some common factors. Future DR prevention and intervention programs may benefit from improving these factors to reduce the risk of DR by stage.

## Background

Diabetic retinopathy (DR) is the most frequent microvascular complication of Type 2 diabetes mellitus (T2DM) and remains a leading cause of preventable visual impairment and blindness in the working-age population worldwide [1, 2]. With the alarmingly highly increasing prevalence of T2DM, the number of DR is expected to grow from 126.6million in 2010 to 191.0million by 2030 globally [3]. China has the largest number of people affected by diabetes which has reached 140.9million in 2021, posing a huge burden of DR [4]. Several epidemiological studies have reported a high prevalence (ranging from 11.9 to 43.1%) of DR among people with diabetes in mainland China, with a higher prevalence in rural areas (29.1%) than in urban areas (18.1%) [5–7].

DR is a progressive disease caused by damage to the blood vessels of the light-sensitive tissue at the retina [2]. Based on its severity, DR can be broadly divided into non-proliferative DR (indicated by microaneurysms, intraretinal hemorrhages, venous beading, intraretinal microvascular abnormalities, etc.) and proliferative DR (characterized by neovascularization, vitreous hemorrhage, preretinal hemorrhage, etc.) [8]. Individuals who progress from non-proliferative DR to proliferative DR frequently experience a decline in best-corrected visual acuity which can result in permanent vision loss, posing a heavy burden on individuals, families, and the society [9]. It has been estimated that the costs of caring for individuals with proliferative DR was four times greater than the costs of managing individuals with non-proliferative DR ($ 1,207 vs. $ 292) [10]. However, DR often has no overt symptoms in the early stage, making it difficult to be detected and treated in time [11]. It is thus crucial to generate a precise and comprehensive estimate of the risk factors of DR by stages in order to guide specific and targeted prevention and intervention for the development and progression of DR and to achieve optimal clinical management of diabetes [7].

A large body of research has been conducted to explore the risk factors of DR among people with T2DM, which can be generally classified into five major types: genetic, disease-related, socioeconomic, behavioral, and psychosocial factors. According to a meta-analysis, several genes (e.g., aldose reductase gene, z-2 microsatellite) have been identified to confer risk or protection in DR [3, 12]. Regarding disease-related factors, metabolic control and disease duration were recognized as the most significant risk factors, explaining about 11% of the risk of developing DR [13]. Several socioeconomic factors have been identified as important determinants of DR risk, with older age, female gender, minority ethnicity, and poor health service being associated with higher prevalence and severity of DR [14–18]. In addition, a range of behavioral factors have been shown to be related to DR, including dietary habits, physical activity, smoking, glycemic and blood pressure control [19, 20]. As for psychosocial factors, better diabetes self-efficacy, social support, and family function were reported to be significantly associated with better diabetes self-management and glycemic control [21, 22], thus reducing the risk of DR [23].

A review of past literature has identified several limitations. First, although a huge gap exists in the prevalence and risk factors of DR between urban and rural areas, relatively less research attention has been directed to the resource-deprived rural areas. Second, although the risk factors may vary according to the different stages of DR, especially for the vision-threatening stages (e.g., proliferative DR) [24], few studies have distinguished between non-proliferative DR and proliferative DR in the exploration of risk factors. Third, although a variety of risk factors have been identified to be associated with DR by previous studies, most studies were not theory-driven and thus unable to provide a comprehensive conceptual framework to better understand the underlying mechanism of DR development and progression.

In light of the above-mentioned limitations, we conducted the current study to investigate and compare the proportion and risk factors of non-proliferative DR and proliferative DR among people with T2DM in less-developed rural areas in Hunan Province of China. Hunan province is representative of Central South China in geography (mixed plains and mountainous areas), climate (humid subtropical), culture, demographics (Han and multi-minorities), economy, health policy, and lifestyle (diet and physical activities) [15]. Specifically, our study was guided by the socio-ecological model, which is one of the most used models to understand risk factors of health and to frame potential prevention strategies [25]. According to this model, health is affected by the interaction between multiple factors, including individual, relationship, and community and societal factors. Based on the socio-ecological model, we hypothesize the following risk factors of DR among people with T2DM: individual factors that include socio-demographic, clinical, psychological, and behavioral characteristics, relationship factors that include social support and family function, and community and societal factors that include accessibility of a range of health services.

## Method

### Study design and setting

A multi-site cross-sectional survey was conducted in the less-developed rural areas in Hunan Province of China from January 2021 to February 2021. Participants were recruited through the Diabetic Retinopathy Screening Program (DRSP), which was a welfare program that provides free DR screening for residents in resource-deprived areas. Four poverty-stricken national counties were selected from Hunan Province, including Jianghua county in Yongzhou city, Huayuan county in Xiangxi Autonomous Prefecture, Sangzhi county in Zhangjiajie city, and Cili county in Zhangjiajie city. The four counties were representative of the less-developed rural areas in Hunan Province, with geographic locations covering the Southern to Northern Hunan, and the proportions of minority ethnicity ranging from 10.24 to 75.28%. The per capita GDP of the four counties ranged from 3848 to 4491 US dollars in 2020, much lower than the average level of China (10,779 US dollars).

### Participants

The participants were recruited from the largest local county-level hospitals in the four counties. The eligibility criteria of the participants included a) ≥ 18 years of age; b) diagnosed with T2DM according to the World Health Organization criteria: fasting capillary blood glucose ≥ 6.1mmol/L or plasma glucose ≥ 7mmol/L and/or 2-hour postprandial blood glucose ≥ 11.1mmol/L; c) resident population that complied with the National Bureau of Statistic criterion, requiring living in the selected rural areas for more than 6 months [26]; d) capable of understanding and communicating in Chinese. Exclusion criteria: (a) already diagnosed with DR; (b) cognitively impaired; (c) with severe mental illness.

Because more formal procedures for sample size determination in multinomial logistic regression are not available. So we first used the standardized rule-of-thumb sample size guideline for multinomial logistic regression requires at least 15 cases for each predictor [27]. And then examined the power of such a sample size assuming a binary logistic regression model for comparing non-diabetes retinopathy versus diabetes retinopathy using G*Power 3 [28]. A total of 11 independent variables were selected in the study based on the socio-ecological model, literature, and clinical experience, indicating a minimum of 165 participants required for the current study. Adjusting for a dropout rate of approximately 20% the required sample size would be about 200. For a single binary risk factor, an odds ratio of 1.4 for a null hypothesis of 0.40 for low risk, an alpha of 0.10 for a one-tailed test, a multiple correlation of 0.25 with other risk factors, and a sample size of 200, the power of the test is 0.75, indicating a sufficient sample size to exam the associating factors.

### Procedure

The study was approved by the Institutional Review Board of the Second Xiangya Hospital, Central South University (No. LYF2022014) and was in accordance with the Declaration of Helsinki. All participants were informed of the purpose, benefits, risks, and significance of the study, gave verbal informed consent, and participated voluntarily. Participants were recruited when they came to the research sites for free diabetic retinopathy screening by the trained research assistants. Participants were approached when they were waiting for DR screening and eligible participants were invited to participate in the study after providing informed consent. The participants were asked to fill in standard questionnaires, with their health records reviewed by the research assistants. The research assistants were available to answer questions and check each questionnaire to avoid unintentional missing items or pages. Each questionnaire took approximately 10–15min to complete. After completing the questionnaires, participants proceeded to their DR screening.

### Measurements

#### Sample characteristics

A researcher-designed questionnaire was used to obtain information on the participants’ socio-demographic and clinical characteristics. Socio-demographic information included age, gender, ethnicity, marital status, educational attainment, occupation status, and household income level, while clinical information included duration of diabetes, history of diabetic nephropathy, and history of hypertension.

#### Diagnosis of DR by stages

Information on DR diagnosis was collected by reviewing the health records from the free diabetic retinopathy screening program. DR was assessed by retinal photography using a 45° CR-DGi NM Fundus Camera. The photographs were digitally stored. Data were digitally recorded and analyzed by three junior ophthalmologists with a minimum of 3 years of experience of the institutes. A senior ophthalmologist expert with a minimum of 5 years of experience was invited to resolve any discrepancies and seek agreement among the junior ophthalmologists. Considering the precise of screening methods, we only identify the stages of DR as Non-proliferative DR and proliferative DR. Of that, non-DR was defined as no presence of apparent retinopathy by ophthalmological examination according to the international clinical diabetic retinopathy and diabetic macular edema disease severity scales [8]. Non-proliferative DR was defined as the presence of microaneurysms, intraretinal hemorrhages, venous beading, or intraretinal microvascular abnormalities, etc., while excluding the signs of proliferative retinopathy [8]. Proliferative DR was defined as the presence of neovascularization, vitreous hemorrhage or preretinal hemorrhage [8]. All participants underwent DR screening by physicians with a minimum of 2 years of experience using fundus camera. Two fundus photographs (the macular fovea and the optical center) of both eyes of each participant had been taken following mydriasis.

#### Diabetes self-efficacy

 Diabetes self-efficacy was measured by the Diabetes Self-Efficacy Scale (DSES, Chinese version) [29]. It was developed by Hurley and Shea in 1992 [30]. The revised 26-item scale assesses self-reported self-care behaviors among T2DM patients. Each item is rated on a 5-point Likert scale from 1 (not at all confident) to 5 (very confident). The total score ranges from 26 to 130, with a higher score indicating a higher level of diabetes self-efficacy. The Cronbach’s α was 0.91, and the test-retest reliability was 0.85 among Chinese people with diabetes [29]. In this sample, the Chinese version of DSES showed good internal consistency with a Cronbach’s α of 0.94.

#### Diabetes self-care behaviors

Diabetes self‐care behaviors were measured by the Summary of Diabetes Self Care Activities Measure (SDSCA, Chinese version) [31]. It was developed by Toobert in 2000 [32]. The revised 11-item SDSCA assesses self-reported self-care behaviors from the following six dimensions: general diet, specific diet, physical activity, self-monitoring of blood glucose, foot care, and medication adherence. Each item is rated on a 5-point Likert scale from 0 (never) to 7 (daily). The total score ranges from 0 to 77, with a higher score indicating a higher level of self-management behaviors. The dimension of “foot care” was excluded from the present study due to irrelevance to the study aims. The Cronbach’s α for the remaining five dimensions was 0.712 ~ 0.971, indicating good reliability of the scale [31]. In this sample, the Chinese version of SDSCA showed good internal consistency with the Cronbach’s α ranging from 0.66 ~ 0.94 for the remaining five dimensions.

#### Social support

Social support was measured by the Social Support Rating Scale (SSRS, Chinese version) [33]. Xiao developed the 10-item scale during 1986–1993 [33]. The scale consists of three dimensions: objective support, subjective support, and utilization degree of social support. A higher score indicates a higher level of social support. The SSRS has demonstrated high internal consistency (Cronbach’s α = 0.92), indicating good reliability [33]. In this sample, the SSRS showed acceptable internal consistency with a Cronbach’s α of 0.72.

#### Family function

Family function was measured by the Family Adaptation, Partnership, Growth, Affection and Resolve (APGAR) index scale. It was developed by Smilkstein in 1978 and contains 5 items to assess respondents’ satisfaction with family function [34]. Each item is rated on a 3-point Likert scale from 0 (hardly ever) to 2 (almost always). The total score ranges from 0 to 10, with a higher score representing higher family function. The Cronbach’s α was 0.86 and the test-retest reliability was 0.80 [34]. In this sample, the APGAR showed good internal consistency with a Cronbach’s α of 0.92.

#### Health service accessibility

Health service accessibility was measured by a self-designed questionnaire with reference to the free health services for T2DM patients based on the third version of National Fundamental Public Health Service Standard in China. It contains 4 “yes-no” questions that asked about respondents’ access to free blood glucose monitoring services, complication prevention and management education, regular fundus examination notice, and lifestyle education.

### Data analysis

Statistical analysis was performed using the SPSS 25.0 software. Variables based on qualitative measurements were presented as frequencies with percentages. Normality distribution of variables based on quantitative measurements was checked by the Shapiro–Wilk test. Means ± standard deviations (SD) were used if data were normally distributed, and medians and interquartile range (IQR) were used if data showed skewed distribution. First, group comparisons were conducted by chi-square test, Fisher’s exact test, or Kruskal-Wallis test depending on variable types and distribution. Bonferroni corrections were used for multiple comparisons, with *p* < 0.05/n (n is the number of subgroups) considered statistically significant. Second, a multinomial logistic regression model was employed to compare the impacts of the risk factors on the non-proliferative DR and proliferative DR groups, using the non-DR group as the reference. The risk factors were selected and included in the model guided by the socio-ecological model and based on the past literature and clinical experience. Finally, a multinomial logistic regression model was used to explore the relative risk factors of proliferative DR, using the non-proliferative DR group as the reference. The level of significance was set to *p* < 0.05.

## Result

### Sample characteristics

A total of 207 participants were included in this study, eleven withdrew from the study due to family emergency, leading to a final sample of 196 participants who completed the study. Table1 shows a summary of sample characteristics. The participants had a median age of 57.00 years (IQR:52.00–65.00), with about half being female (53.6%) and minority groups (48.5%). Most were married (89.8%) and with less than 9 years of educational attainment (60.2%). About one-third (36.7%) were farmers and 15.8% had a low annual household net income below the national poverty line ($ 361) in China. The median duration of diabetes was 11.00 (IQR:7.00–15.00), and 10.2% had a history of diabetic nephropathy, while 38.3% had a history of hypertension. Details on participants’ diabetes self-efficacy, diabetes self‐care behaviors, social support, family function, and health service accessibility were shown in Table1.

**Table 1:** Characteristics of the non-DR, non-proliferative DR, and proliferative DR groups

Variables		Total (n = 196)	Non-DR (n = 79)	Non-proliferative DR (n = 73)	Proliferative DR (n = 44)	H/***χ***^***2***^	***p***-Value	Post-hoc test*
**Socio-demographic and clinical characteristics**								
Age	≤ 60 years	124 (63.3)	51 (64.6)	42 (57.5)	31 (70.5)	2.067	0.356^a^	NA
	≤ 60 years	72 (36.7)	28 (35.4)	31 (42.5)	13 (29.5)			
Sex	Female	105 (53.6)	42 (53.2)	34 (46.6)	29 (65.9)	4.135	0.127^a^	NA
	Male	91 (46.4)	37 (46.8)	39 (53.4)	15 (34.1)			
Ethnicity	Han	**101 (51.5)**	**40 (50.6)**	**30 (41.1)**	**31 (70.5)**	**9.517**	**0.009** ^**a**^	**NP**
	Minority	95 (48.5)	39 (49.4)	43 (58.9)	13 (29.5)			
Marital status	Married	176 (89.8)	70 (88.6)	68 (93.2)	38 (86.4)		0.447^c^	NA
	Unmarried, widows, divorces and separations	20 (10.2)	9 (11.4)	5 (6.8)	6 (13.6)			
Educational attainment (years)	≤ 9	118 (60.2)	46 (58.2)	41 (56.2)	31 (70.5)	2.556	0.279^a^	NA
	> 9	78 (39.8)	33 (41.8)	32 (43.8)	13 (29.5)			
Occupation status	Famer	72 (36.7)	26 (32.9)	28 (38.4)	18 (36.7)	0.909	0.635^a^	NA
	Others	124 (63.3)	53 (67.1)	45 (61.6)	26 (59.1)			
Low-income family	No	165 (84.2)	66 (83.5)	64 (87.7)	35 (79.5)	1.402	0.496^a^	NA
	Yes	31 (15.8)	13 (16.5)	9 (12.3)	9 (20.5)			
Diabetes duration (years)		**11.00 (7.00–15.00 )**	**9.00 (3.00–11.00 )**	**11.00 (8.00–16.00 )**	**11.00 (9.00–19.00 )**	**16.662**	**<0.001** ^**b**^	**NW, PW**
History of diabetes nephropathy	No	**176 (89.8)**	**76 (96.2)**	**63 (86.3)**	**37 (84.1)**		**0.034** ^**c**^	NA**
	Yes	20 (10.2)	3 (3.8)	10 (13.7)	7 (15.9)			
History of hypertension	No	121 (61.7)	52 (65.8)	44 (60.3)	25 (56.8)	1.075	0.584^a^	NA
	Yes	75 (38.3)	27 (34.2)	29 (39.7)	19 (43.2)			
**Diabetes self-efficacy**		**101.00 (84.00–116.00 )**	**101.00 (81.00–113.00 )**	**109.00 (95.00–122.00 )**	**92.00 (77.00–104.00 )**	**13.893**	**0.001** ^**b**^	**NW, NP**
**Behavioral characteristics**								
General diet		8.00 (7.00–12.00 )	8.00 (7.00–11.00 )	9.00 (7.00–12.00 )	8.00 (7.00–12.00 )	2.408	0.300^b^	NA
Specific diet		14.00 (8.00–14.00 )	14.00 (8.00–14.00 )	14.00 (9.00–14.00 )	14.00 (9.00–14.00 )	0.340	0.844^b^	NA
Physical activity		**7.00 (4.00–13.00 )**	**7.00 (5.00–14.00 )**	**8.00 (6.00–14.00 )**	**6.00 (0.00–8.00 )**	**13.273**	**0.001** ^**b**^	**PW, NP**
Self-monitoring of blood glucose		**4.00 (2.00–12.00 )**	**2.00 (0.00–7.00 )**	**6.00 (2.00–14.00 )**	**6.00 (2.00–14.00 )**	**11.917**	**0.003** ^**b**^	**NW, PW**
Medication adherance		7.00 (7.00–7.00 )	7.00 (7.00–7.00 )	7.00 (7.00–7.00 )	7.00 (7.00–7.00 )	1.924	0.382^b^	NA
**Social support**		38.00 (31.00–44.00 )	38.00 (31.00–44.00 )	39.00 (32.00–44.00 )	37.00 (31.00–43.00 )	0.893	0.640^b^	NA
**Family function**		**10.00 (6.00–10.00 )**	**10.00 (5.00–10.00 )**	**10.00 (8.00–10.00 )**	**8.00 (5.00–10.00 )**	**8.14**	**0.017** ^**b**^	**NP**
**Health service**								
Accessible free blood glucose monitoring service	No	77 (39.3)	34 (43.0)	26 (35.6)	17 (38.6)	0.886	0.642^a^	NA
	Yes	119 (60.7)	45 (57.0)	47 (64.4)	27 (61.4)			
Accessible complication prevention and management education	No	78 (39.8)	29 (36.7)	29 (39.7)	20 (45.5)	0.902	0.637^a^	NA
	Yes	118 (60.2)	50 (63.3)	44 (60.3)	24 (54.5)			
Accessible undergoing regular fundus examination notice	No	107 (54.6)	43 (54.4)	34 (46.6)	30 (68.2)	5.171	0.075^a^	NA
	Yes	89 (45.4)	36 (45.6)	39 (53.4)	14 (31.8)			
Accessible lifestyle health education	No	153 (78.1)	61 (77.2)	61 (83.6)	31 (70.5)	2.809	0.245^a^	NA
	Yes	43 (21.9)	18 (22.8)	12 (16.4)	13 (29.5)			

^a^ Chi-square test; ^b^ Kruskal-Wallis test; ^c^ Fisher’s exact test;

Abbreviations: NA, not applicable; NW, non-proliferative DR group versus non-DR group; PW, proliferative DR group versus non-DR group; PN, proliferative DR group versus non-proliferative DR group

* *p* < 0.05/3 = 0.0167

**non-proliferative DR group versus non-DR group: *p* = 0.041≤0.0167, proliferative DR group versus non-DR group: *p* = 0.034≤0.0167, proliferative DR group versus non-proliferative DR group, *p* = 0.790≤0.0167

### Comparisons of sample characteristics by DR status

Based on the health records of DR screening, a total of 59.6% (n = 117) of the participants were identified as DR, including 37.2% (73/196) of non-proliferative DR and 22.4% (44/196) of proliferative DR. Table1 shows comparisons of socio-demographic and clinical characteristics, diabetes self-efficacy, diabetes self‐care behaviors, social support, family function, and health service accessibility among the non-DR group, non-proliferative DR group, and proliferative DR group.

For socio-demographic and clinical characteristics, significant group differences were found in ethnicity, diabetes duration, and history of diabetic nephropathy. Multi-group comparisons revealed that the proportion of minorities in the non-proliferative DR group was significantly higher than that in the proliferative DR group (70.5% vs. 41.1%, *p*<0.05). The median diabetes duration of the proliferative DR/ non-proliferative DR group was significantly higher than that of the non-DR group [non-DR: 9.00 (3.00–11.00 ), non-proliferative DR: 11.00 (8.00–16.00 ), proliferative DR: 11.00 (9.00–19.00 ); *p* < 0.05]. Although a significant difference in the history of diabetes nephropathy was found among the three groups, no significant difference was found between any two sub-groups after multiple testing.

Among the other risk factors included, significant group differences were found in diabetes self-efficacy, diabetes self‐care behaviors, and family function, but not social support, or health service accessibility among the three groups. The score of diabetes self‐efficacy was significantly higher in the non-proliferative DR group than in the proliferative DR/ non-DR group [non-DR: 101.00 (81.00–113.00 ), non-proliferative DR: 109.00 (95.00–122.00 ), proliferative DR: 92.00 (77.00–104.00 ); *p* < 0.05]. For diabetes self‐care behaviors, significant differences were found in physical activity and self-monitoring of blood glucose among the three groups. The score of physical activities was significantly lower in the proliferative DR group than in the non-DR/non-proliferative DR group (non-DR: 7.00 (5.00–14.00 ), non-proliferative DR: 8.00 (6.00–14.00 ), proliferative DR: 6.00 (0.00–8.00 ); p<0.05). The score of self-monitoring of blood glucose was significantly lower in the non-DR group than in the non-proliferative DR/ proliferative DR group (non-DR: 2.00 (0.00–7.00 ), non-proliferative DR: 6.00 (2.00–14.00 ), proliferative DR: 6.00 (2.00–14.00 ); p<0.05). Furthermore, the score of family function was significantly higher in the non-proliferative DR group than in the proliferative DR group (non-proliferative DR 10.00 (8.00–10.00 ), proliferative DR 8.00 (5.00–10.00 ); p < 0.05).

### Risk factors of DR by stages

Table2 shows the risk factors of non-proliferative DR and proliferative DR groups, using the non-DR group as the reference. Compared to the non-DR group, the risk factors of non-proliferative DR and proliferative DR were diabetes duration (OR: 1.10, 95 CI%: 1.04–1.17; OR: 1.14, 95 CI% 1.06–1.22), self-monitoring of blood glucose (OR: 1.09, 95 CI% 1.01–1.17; OR: 1.11, 95 CI%: 1.02–1.20) (*p*<0.05); the protective factor of non-proliferative DR was accessible complication prevention and management education (OR: 0.37, 95 CI% 0.14–0.94) while the protective factors of proliferative DR were physical activities (OR: 0.89, 95 CI%: 0.80–0.98) (*p*<0.05).

**Table 2:** Results of the analysis investigating the association between the factors and non-proliferative DR/proliferative DR status using multinomial logistic regression models

Variables	Non-proliferative DR^a^			Proliferative DR^a^		
	OR	95% CI	*p*-Value	OR	95% CI	*p*-Value
Ethnicity (ref. Han)	1.64	(0.79, 3.40)	0.184	0.72	(0.30, 1.78)	0.482
**Diabetes duration (years)**	**1.10**	**(1.04, 1.17)**	**0.002**	**1.14**	**(1.06, 1.22)**	**<0.001**
History of diabetes nephropathy (ref. No)	3.40	(0.81, 14.14)	0.094	2.78	(0.60, 12.82)	0.189
Diabetes self-efficacy	1.02	(0.99, 1.04)	0.15	1.00	(0.98, 1.02)	0.911
**Physical activity**	1.00	(0.92, 1.09)	0.969	**0.89**	**(0.80, 0.98)**	**0.020**
**Self-monitoring of blood glucose**	**1.09**	**(1.01, 1.17)**	**0.02**	**1.11**	**(1.02, 1.20)**	**0.013**
Family function	1.07	(0.93, 1.24)	0.357	0.90	(0.78, 1.04)	0.159
Accessible free blood glucose monitoring service(ref. No)	1.56	(0.71, 3.40)	0.265	1.48	(0.58, 3.78)	0.417
**Accessible complication prevention and management education(ref. No)**	**0.37**	**(0.14, 0.94)**	**0.037**	0.72	(0.23, 2.21)	0.560
Accessible to undergoing regular fundus examination notice (ref. No)	1.76	(0.72, 4.34)	0.216	1.13	(0.39, 3.29)	0.828
Accessible to lifestyle education (ref. No)	1.51	(0.52, 4.36)	0.445	0.59	(0.19, 1.83)	0.363

^a^ The non-DR group was used as the reference

Abbreviations: CI, confidence interval; OR, odds ratio

Table3 shows the risk factors of proliferative DR, using the non-proliferative DR group as the reference. Compared to the non-proliferative DR group, the protective factors of proliferative DR were physical activities (OR: 0.89, 95 CI% 0.02–0.89) and family function (OR: 0.84, 95 CI%: 0.04–0.84) (*p*<0.05).

**Table 3:** Results of the analysis investigating the association between the factors and proliferative DR status using multinomial logistic regression models

Variables	Proliferative DR^a^		
	OR	95% CI	*p*-Value
Ethnicity (ref. Han)	0.44	(0.07, 0.44)	0.072
Diabetes duration (years)	1.03	(0.29, 1.03)	0.290
History of diabetes nephropathy (ref. No)	0.82	(0.75, 0.82)	0.751
Diabetes self-efficacy	0.98	(0.17, 0.98)	0.170
**Physical activity**	**0.89**	**(0.02, 0.89)**	**0.021**
Self-monitoring of blood glucose	1.02	(0.64, 1.02)	0.643
**Family function**	**0.84**	**(0.04, 0.84)**	**0.039**
Accessible to free blood glucose monitoring service (ref. No)	0.95	(0.91, 0.95)	0.911
Accessible to complication prevention and management education (ref. No)	1.96	(0.26, 1.96)	0.255
Accessible to undergoing regular fundus examination notice (ref. No)	0.64	(0.42, 0.64)	0.424
Accessible to lifestyle education (ref. No)	0.39	(0.12, 0.39)	0.115

^a^ The non-proliferative DR group was used as the reference

Abbreviations: CI, confidence interval; OR, odds ratio

## Discussion

This study showed a high proportion of DR among T2DM patients in less-developed rural areas in Hunan Province of China that reached approximately 60%. The associated risk factors of DR identified in this study provided empirical evidence to the socio-ecological model. Compared to the non-DR group, diabetes duration and self-monitoring of blood glucose were the risk factors of non-proliferative DR and proliferative DR. In addition, accessible complication prevention and management education was the protective factors of non-proliferative DR, while physical activity was the protective factors of proliferative DR. Compared to non-proliferative DR, physical activity and family function were the protective factors of proliferative DR. These results may contribute to the development of targeted DR prevention and intervention programs in rural primary care settings.

The proportion of DR (DR: 59.6%, non-proliferative DR:37.2%, and proliferative DR: 22.4%) reported in this study among T2DM patients in less-developed rural areas in Hunan Province of China was higher than that reported in a meta-analysis that included diabetes patients in both urban and rural China (DR: 18.45%, non-proliferative DR: 15.06% and proliferative DR: 0.99%) [35]. This is consistent with evidence showing a higher proportion of DR among T2DM patients in rural areas than in urban areas [5] and may be explained by the much lower health services accessibility and lower health literacy in the rural areas.

Regarding associating factors of DR by stage, our study has identified the following five factors of DR (including either non-proliferative DR or proliferative DR or both): diabetes duration, self-monitoring of blood glucose, physical activity, family function, and accessible complication prevention and management education. The finding that diabetes duration was the risk factors of non-proliferative DR and proliferative DR for T2DM patients was consistent with previous research among T2DM patients [36]. This may be explained by the chronic hyperglycemia due to longer diabetes duration, which increased vascular permeability and microvascular occlusion of the retina, leading to DR [37]. The finding that T2DM patients with non-proliferative and proliferative DR took more frequent self-monitoring of blood glucose than those without DR may be explained by the worse glucose control of non-proliferative or proliferative DR than those without DR [11]. As a result, they were often asked to self-monitor blood glucose more frequently, especially when they need insulin therapy [38].

With regard to those different associating factors, first, accessible complication prevention and management education was found to be the protective factors of non-proliferative DR. It could be explained that diabetes-related health services can promote positive self-management behaviors for T2DM patients, which could improve glycemic control and minimize the development of complications [39]. However, this association was not found for proliferative DR, indicating the health service may play a more important role in the early development of DR than the progression of DR [40]. In addition, compared to the non-DR or non-proliferative DR group, physical activities were found to be the protective factors of proliferative DR. This finding was consistent with previous studies showing more physical activity was associated with decreased risk of proliferative DR [41]. It may be explained by the better glycemic control and improved endothelial function associated with more physical activities could reduce the risk of proliferative DR [42]. However, our study did not find the association between physical activity and non-proliferative DR, although some studies revealed that physical activity was the protective factor of severe non-proliferative DR [43, 44]. It may be because the severity of non-proliferative DR was not clarified in this study; thus, this association failed to be obtained. Moreover, our study showed that family function was the protective factors of proliferative DR among T2DM patients as compared to non-proliferative DR but this association was not found for non-proliferative DR. Generally speaking, T2DM patients with better family function may have better self-management, and thus reducing the progression of DR [45]. However, patients often experience a decline in visual acuity as the progression of DR and then causing poor self-care ability. Thus, people with developed DR may rely more on family support and resources than those without DR [27].

### Limitations

This study has some limitations that should be noted. First, this study was a cross-sectional survey, and we cannot derive any conclusions on the causality of the associations. Second, participants were recruited from the free DR screening program, which may attract more health conscious T2DM patients or T2DM patients with ocular discomfort, thus overestimating the proportion of DR. Third, recall bias and biased responses in self-reported diabetic self-care behaviors (e.g., dietary intake and the frequency of self-monitoring of blood glucose) in this study may exist, and some participants might not provide correct information. Fourth, although we selected a wide range of risk factors of DR based on the social-ecological model and literature, some potentially associating factors (e.g., the duration of diabetic nephropathy and proteinuria) were not included in this study. Finally, although non-proliferative DR and proliferative DR can be divided into mild NPDR, moderate NPDR, severe NPDR, early PDR, fibrous proliferation, and advanced PDR, we only identified the risk factors of refined stages of non-proliferative DR or proliferative DR due to the limited time and budget to recruit enough sample size.

### Implication

This study provides the following insights and guidance for future research and clinical practice. First, the high proportion of DR found in the current study implies that routine DR screening is highly recommended in less-developed rural areas to facilitate early diagnosis and timely intervention. However, rural areas are often confronted with objective challenges such as inconvenient transportation and lack of access to the screening equipment, which may prevent diabetes patients from having DR screening [46]. This indicates that more health resources should be provided to the rural areas such as free diabetes self-management education programs and free DR screening programs. Second, the risk factors of DR identified in the study provide empirical evidence for developing DR prevention and intervention programs. For instance, diabetes patients should be encouraged to engage in more physical activities and provided with early education on preventing and managing diabetes-related complications. Also, family members of diabetes patients should be involved in managing of diabetes to provide care, support, and encouragement, especially for those patients with early stages of DR. All these measures may effectively reduce the diabetes patients’ risk of developing DR or progressing from non-proliferative DR to proliferative DR.

## Conclusion

Our study showed that DR was prevalent among T2DM patients in less-developed rural areas, which warrants research and clinical attention and indicates the need of strengthening DR screening. Risk factors of DR by stages included longer diabetes duration, more frequent self-monitoring of blood glucose, fewer physical activities, worse family function and inaccessible complication prevention and management education. Future DR prevention and intervention programs may benefit from improving these factors to reduce the risk of DR development and progression.

## Data Availability

The datasets generated and analyzed will not be publicly available due to the confidentiality agreement with the participants. The data will be however available from the corresponding author on reasonable request.

## Abbreviations

T2DM: type 2 diabetes mellitus.
DR: diabetic retinopathy.
IQR: interquartile range.
